# Test–retest reliability and agreement of remote home-based functional capacity self-administered assessments in community-dwelling, socially isolated older adults

**DOI:** 10.1177/20552076241254904

**Published:** 2024-05-21

**Authors:** Rodrigo Villar, Thomas Beltrame, Gabriela Ferreira dos Santos, Anderson Saranz Zago, Danilo Sales Bocalini, Francisco Luciano Pontes Júnior

**Affiliations:** 1 Cardiorespiratory & Physiology of Exercise Research Laboratory, Faculty of Kinesiology and Recreation Management, 8664 University of Manitoba, Winnipeg, MB, Canada; 2 Department of Physical Therapy, 67828 Federal University of São Carlos, São Carlos, São Paulo, Brazil; 3 Department of Physiotherapy, Universidade Ibirapuera, 28133 São Paulo, São Paulo, Brazil; 4 Institute of Computing, University of Campinas, Campinas, São Paulo, Brazil; 5 Physiology of Exercise & Aging Laboratory, School of Arts, Sciences and Humanities, University of São Paulo, São Paulo, Brazil; 6 Department of Physical Education, Graduate Program in Movement Sciences, São Paulo State University-UNESP, SP, Brazil; 7 Biochemistry and Physiology Laboratory. Physical Education and Sport Center of Federal University of Espirito Santo, Vitoria, Brazil

**Keywords:** Functional capacity, self-assessment, remote administration, home-based-testing, aging

## Abstract

**Objectives:**

To determine the test–retest reliability and agreement of home-based functional capacity self-administered assessments in socially isolated older adults.

**Methods:**

Fourteen community-dwelling older adults (eight females, 67.9 ± 7.7 years) volunteered for this study. Before testing, participants were screened online for eligibility and received instructional videos explaining test set-up and execution. Participants underwent the 30-second sit-to-standing test, gait speed tests at the usual pace, and timed-up-and-go tests administered 4 weeks apart. For the 30-second sit-to-standing protocol, participants were instructed to repeatedly sit and stand from a chair (with a height of ∼ 43 cm and without armrests) for 30 s, with the number of repetitions recorded. In the gait speed test protocol, participants were instructed to walk at their usual and comfortable pace, with the time taken recorded (seconds). In the timed-up-and-go, participants stood up from a chair, walked as fast as possible for 3 m, circled a marked point, and returned to the chair to sit down, completing the test, with the score recorded (seconds). A trained researcher conducted the scoring virtually via synchronous video.

**Results:**

30-second sit-to-standing, gait speed test, and timed-up-and-go showed excellent mean coefficient of variation values (2.0–4.9), small standard error of measurement (0.08–1.27), excellent intraclass coefficient (0.97–0.99), very strong correlations (0.97–0.99) and good agreement between the two days of testing.

**Conclusion:**

Self-administered functional capacity tests performed by older adults at home were reliable with good agreement. Healthcare professionals and older adults should take advantage of simple remote self-administered assessments performed at home to evaluate older adults’ health status.

## Introduction

Given that socially isolated older adults are more vulnerable and at higher risk of accelerated decline in functional capacity and potentially more limited in accessing health services, establishing whether remote home-based functional capacity tests are reliable could inform strategies to monitor functional capacity status, changes over time, and access the effectiveness of interventions (e.g. exercise, diet, or both combined). Many older adults live in socially isolated settings such as institutions (e.g. nursing homes, retirement homes, and senior homes) or their homes. These numbers increased dramatically during the COVID-19 pandemic due to social isolation strategies (e.g. lockdown and social restrictions) aimed at minimizing its spread. Particularly, vulnerable populations, including older adults at risk of falling, frail individuals, and people living with chronic diseases^1,2^ were more likely to experience social isolation. As a result, they were more exposed to harmful health consequences such as sedentary behaviors and low-energy expenditure activities (i.e. sitting, lying, and reading). The lack of physical activity increases their risk of chronic diseases, falls and fractures, sarcopenia, frailty, hospitalization, comorbidities, and mortality.^3^ Therefore, maintaining a healthy, physically active lifestyle for those experiencing social isolation is critical for their well-being and quality of life.

Physical activity is a non-pharmacological tool recommended for preventing, treating, and controlling associated risks for developing chronic diseases^4,5^ and improving mental health.^6^ The World Health Organization^7^ reported that physical activity decreases the incidence of cardiovascular and cerebrovascular diseases, diabetes, cancer, and excessive body fat in older adults. Benefits include increased strength and muscle mass, improved balance, maintenance or improvement of functional capacity, lower fall risk, and reduced cognitive decline. Physical activity can also be an efficient, easy, and cost-effective way to counter the decline in functional capacity with aging and mitigate the impact on health caused by social isolation and sedentary behaviors, as well as reduce adverse health outcomes and physical deconditioning exacerbated by the COVID-19 pandemic.^8,9^

Emerging from the pandemic, there is a new tendency to shift from supervised to remote assessment using tests requiring minimal equipment and space.^10^ Previous studies have shown that supervised functional capacity assessments are reliable in clinical and home settings. Recently, studies have shown remote assessment applications in different populations, including community-dwelling older adults,^11–13^ people living with mobility disorders,^14^ mental health conditions,^15^ diabetes mellitus type 2,^16^ and cancer.^17^ Web-based technologies using asynchronous and synchronous instructions have proven feasible and acceptable in community-dwelling older adults after minor injury^18^ and in frail older adults,^19^ becoming a promising opportunity that should be further explored.^13^ Research has also examined the reliability of remote physical performance compared to face-to-face measurements in older adults, demonstrating high to very high levels of intra-and inter-observer relative reliability.^20^ Despite the great potential of remote assessments, evidence regarding the reliability and agreement of functional capacity and fitness assessments is still scarce.^13,21^ Reliability and agreement studies provide information about the inherent error in measurements, scales, or diagnostic tools.^22^ These analyses are critical for developing appropriate assessments, ensuring quality assurance, and conducting clinical studies safely.^23,24^

Determining functional capacity in clinical or research environments requires financial resources and site visits, increasing the transportation burden for participants. This burden may exclude vulnerable populations (older adults, people living with frailty, and mobility issues), people living in rural areas, or individuals who are unable or unwilling to travel for a research study.^25^ It can also present a challenge for health professionals who are providing home assessments. An alternative to working around this problem is to develop remote home-based functional capacity self-administered assessments that can be performed without in-person professional supervision. This is particularly important for socially isolated older adults because they are more susceptible to functional decline and have less access to the health care system, physical activity, exercise programs, and safe spaces. Although self-administered assessments seem to be a promising alternative to measure functional capacity, it is unclear whether these self-assessments are suitable for home settings and remote synchronous administration. In this context, test-retest reliability and agreement measurements are essential in studies exploring self-administered assessment strategies. Therefore, this study aimed to determine whether participants’ functional capacity would be reliable and in agreement when performed remotely, online, and synchronously at home without the presence of a healthcare professional. We hypothesized that all the selected self-administered functional capacity tests performed at home would demonstrate reliability and agreement between two different testing days in this population.

## Methodology

### Participants

Fourteen older adult volunteers (eight females and six males) underwent self-administered functional capacity tests at home, remotely and synchronously through videoconferencing, without in-person supervision, on 2 days 4 weeks apart. Participants were recruited from the University Medical Center and University Senior Program using a convenient sampling method. Before enrollment in this study, all participants received medical clearance, and the principal investigator addressed any questions and concerns they had. Subsequently, participants signed an informed consent form approved by the University of Sao Paulo Institutional Committee of Ethics in Human Research (protocol CEP-EACH/USP 74029) in accordance with CNS resolution 466/2012.

Older adults with conditions such as dementia, psychiatric disorders, cognitive impairment, stroke, cardiovascular and respiratory diseases, and skeletal muscle impairments that might have limited their ability to perform the functional capacity assessments safely were excluded from the study. Older adults of both sexes (60+ years of age) with independent locomotion, no cognitive impairment (Mini-Mental State),^26^ able to perform the functional capacity tests, possessed an email address, had access to a digital device with a webcam (e.g., cell phone, tablet, laptop, and desktop), and had an internet connection were included in the study. A trained gerontologist collected clinical information (standard medical and health screening) through an online interview to ensure that participants met the eligibility criteria.

### Experimental design

This intra-rater test-retest reliability study design used the Guidelines for Reporting Reliability and Agreement Studies (GRRAS) as a reference for creating this manuscript.^22^ Community-dwelling older adults’ functional capacity assessments were conducted remotely and synchronously through videoconferencing without in-person supervision between April and July 2020 by a trained gerontologist researcher. The order of tests was randomized and counterbalanced across participants. At least one week before testing, participants received three instructional videos explaining in detail how to set up and perform the 30-second sit-to-stand (STS) test, gait speed test (GST), and timed-up-and-go (TUG) test. These videos were sent via email or messaging platforms like WhatsApp, Zoom, or Teams. Participants were encouraged to reach out to the researcher directly via phone, text message, or video chat if they had any questions or concerns before the scheduled test day. Each participant indicated their preferred videoconferencing software (WhatsApp, Google Meet, Zoom, and Teams) during the phone recruitment screening.

Participants were instructed to wear comfortable clothing and shoes, perform the test on a non-slip hard surface with adequate space, refrain from consuming alcohol and caffeinated beverages, avoid vigorous exercise for 24 h prior to testing, and have a substantial meal no later than 2 h before testing to minimize these factors mentioned above that could affect individuals test performance.

The selected tests met specific criteria: (1) designed for older adults, (2) simplicity (easy to understand and perform), (3) quick to execute, (4) required minimal equipment and space, (5) test results associated with health outcomes, and (6) demonstrated high intratester and intertester reliability and agreement in-home or clinical settings. Therefore, the STS, GST, and TUG were used to determine functional capacity. The repeatability and agreement of tests were determined using a paired T-test, coefficient of variation (CV), Bland-Altman plots, and intraclass correlation coefficient (ICC). During the test sessions, the researcher recorded repetitions and time remotely in real-time via videoconferencing while participants performed the tests.

The sample size calculation was based on the study of Peyrusqué et al.^13^ using ICC values from the STS, GST, and TUG assessments. We set the minimally acceptable reliability ICC range of 0.80–0.90 because it represents good or excellent ICCs. The significance level was set at *p *< 0.05 (two-tailed), and we sought a statistical power of 0.80. The number of repetitions per participant was set at 2 (day 1 and day 2), as described by Walter et al.^27^ According to these established parameters, the required sample size was determined to be 14 participants. The effect size for the test–retest reliability was calculated using Cohen's *d*.^28^ The calculated Cohen's *d* value was −0.14 for STS, 0.10 for GST, and −0.07 for TUG.

### Anthropometric assessments

Participants’ body mass was measured to the nearest 0.1 kg using a scale. Their height was measured to the nearest 0.1 cm using a stadiometer attached to the scale (W200A-LED, Welmy, Sao Paulo, Brazil). Body mass index (BMI) was calculated by dividing body mass (in kilograms) by the square of height (in meters) (BMI = body mass (kg)/height (m^2^)). Anthropometric measurements were obtained from each participant´s medical record since they were recruited from a medical facility.

### Remote home-based functional capacity self-administered assessments

The researcher responsible for data collection was trained on all tests to minimize bias. Standardized video instructions were developed and sent to participants at least one week before the testing day. They received three instructional videos explaining in detail how to set up and perform the 30-second STS test, GST, and TUG test. The instructions covered chair height and how to measure it vertically (∼43 cm), floor surface (non-slip hard surfaces and not carpet), and equipment (measurement tape and tape). Individuals were instructed to perform the test in a clear space, ensuring correct movement execution in the same area, with the same equipment, and around the same time as on the first testing day. On the testing day, participants received a meeting link via email and/or WhatsApp, which they accessed on their cell phone, tablet, or computer.

Before starting the test, the researcher reviewed the protocols and procedures with the participants. Internet connectivity and webcam positioning were checked to ensure optimal viewing of the test execution perpendicular to the axis of the task. A wide-angle field of view was selected to capture a broad view of the home space and participants’ complete body movements in a side view without requiring precise camera placement, thereby ensuring more comfort and privacy. Participants conducted familiarization trials for each test under the guidance and validation of the researcher via videoconferencing. Real-time feedback was provided if necessary, and adequate recovery time was allowed after familiarization to prevent any carry-over effects.

Participants were instructed to perform the tests only if someone (spouse or family member) was at home to assist them in case of any unforeseen safety concerns, with strict instructions not to interfere with the test execution. No incidents were reported during both testing days, and none of the participants reported using assistive devices during their daily living activities. All participants demonstrated an understanding of and ability to perform the tests accurately.

#### Sit-to-stand test

The 30-second STS was selected as it measures lower limb muscular endurance and strength and is part of the Senior Fitness Test Protocol.^29^ Also, our group has previous experience using this test.^30^ Briefly, the STS protocol consisted of repeatedly sitting and standing from a chair with a height of ∼ 43 cm, without armrests, for 30 s. The chair was positioned against a wall and stabilized for safety. Participants were required to have their feet flat on the floor, shoulder-width apart, and sit in the middle of the seat with their arms close to their chest and crossed at the wrists. Participants stood upright with their hips and knees fully extended and returned to the seated position, repeating this cycle for 30 s. The total number of completed repetitions was reported as their final score, with successful repetitions defined as those in which the participant stood up completely with full hip and knee extension and returned to the chair correctly with their back making contact with the chair backrest. Scores were categorized as below average, average, and above average based on recommended ranges for STS.^31^ The sit-and-raise tests for musculoskeletal assessment have shown significant predictive value for mortality and risk of falls in older adults.^32^ Hansen et al.^33^ reported intra-rater and agreement with ICC of 0.94 (lower limit 95% CI: 0.90) and standard error of measurement (SEM) of one repetition.

#### Gait speed test

The GST assessed the participant's walking speed. They were instructed to mark the flat hard surface floor with tape to designate the starting and ending lines 6 m apart (4 m for the testing zone and 1 m before and after to allow for acceleration and deceleration). Participants were also instructed to leave a clear, straight path free from any object that could compromise safety and measurement accuracy. The test began when participants started walking at their usual and comfortable pace from the start line and ended when they reached the final line. The timer started when the participants’ first foot crossed the 0-meter line and stopped with the first foot crossing the 4-meter line. Participants performed this test twice, and the best time to complete the distance was considered the final score, expressed in meters.second^−1^ (GST = distance (m)/time (s)). GST results are associated with general health status and serve as an indicator of physiological reserve in older adults. It has also been used as a prognostic factor for the risk of falls, frailty, institutionalization, and mortality in geriatric patients.^34^ In a systematic review by Peel et al.,^35^ older adults exhibited an average gait speed at the usual pace of 0.47 ms^−1^ (95% CI: 0.34–0.57). High interrater and test–retest reliability has been demonstrated for GST.^35^ Kim et al.^36^ reported test–retest reliability with ICC values of 0.72 at the usual pace and found no statistical differences between the two GSTs. Goldberg and Schepens^37^ showed that mean gait speed measurement error values were < 5% and minimal detectable change (MDC) < 13% in community-dwelling older adults.

#### Timed-up-and-go test

The TUG measures dynamic balance, agility, and functional mobility.^38^ Participants were instructed to use a ∼43 cm chair with back support and armrests and mark the no-slip floor's hard surface 3 m apart. Before the test, participants sat down with their backs and arms resting on the chair. The test began with participants standing up and walking as fast as possible for 3 m. Then, they walked around a marked point (tape, similar to using a cone, pillow, or other marker) and finally walked back to the chair and sat down to complete the test. The time taken to complete the test was recorded as the participant's final score. Previous studies reported good inter-observer reliability for the TUG, with ICCs of 0.99 (95% CI: 0.98–0.99) with a mean difference of 0.4 ± 0.6 s, as well as ICC of 0.96 (95% CI: 0.87–0.99) with a mean difference of 0.4 ± 0.4 s between tests.^39,40^ Marques et al.^40^ reported a minimal detectable change (MDC) of 1.84 s for intra-rater reliability. Mesquita et al.^41^ showed ICC values ranging from 0.85 to 0.98, a standard error of ∼1.6 s, and MDC_95%_ (MDC at the 95% CI) of ∼4.5 s within-day test–retest reliability.

### Statistical analysis

Data are presented as means ± standard deviation (SD). The normal distribution of the data was tested using the Shapiro-Wilk normality test, and equal variance using the Brown-Forsythe equal variance test. The repeatability of STS, GST, and TUG was determined as follows: (1) we conducted a paired *T*-tests to detect statistically significant differences between the 2 days of testing (day 1 and day 2), (2) we calculated the coefficient of variation (CV) as (standard deviation/mean)*100 to determine relative variability or dispersion for each participant at each time point, (3) we used Bland-Altman plots to assess agreement the between tests, (4) we calculated the Pearson product-moment correlation coefficient to assess the relationships between the tests, and (5) we computed the ICC to evaluate the reliability of these measurements on both days. The magnitudes of CVs were characterized as excellent (< 10%), good (10%–20%), acceptable (20%–30%), and poor (> 30%).^42^ The Pearson product-moment correlation coefficient (*r*) was considered very strong (*r *= 0.90–1.00), strong (*r *= 0.70–0.89), moderate (*r *= 0.40–0.69), weak (*r *= 0.10–0.39), and negligible (*r *= 0.00–0.10).^43,44^ For ICC, values were classified as excellent (> 0.90), good (0.90–0.75), moderate (0.75–0.50), or poor (<0.50).^45^ The significance level for all statistical analyses was set at 5% (*p *< 0.05). Data were analyzed using Graph Pad Prism 8 (Dotmatics, Boston, MA, USA).

## Results

**Figure 1.**
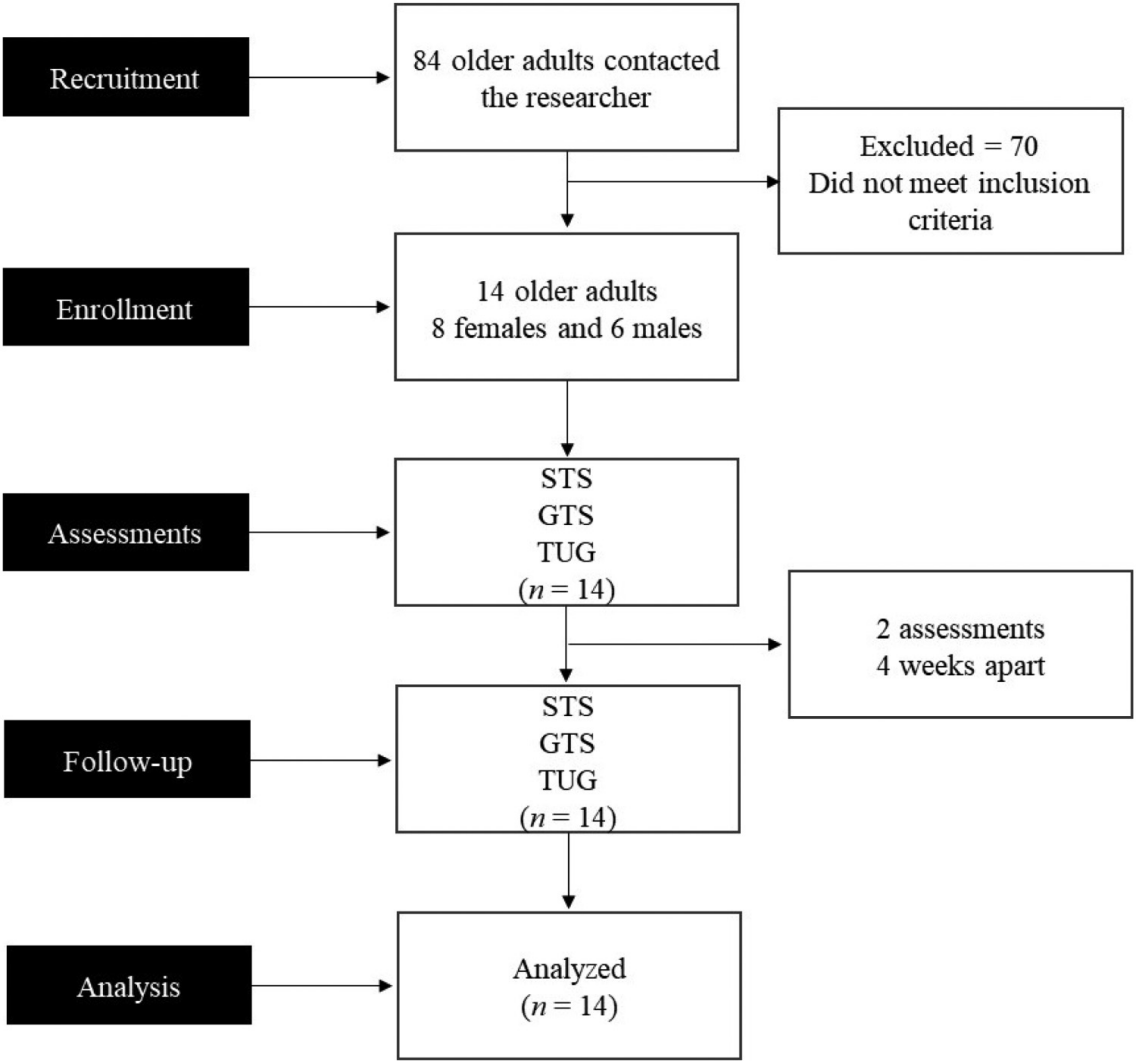
Study flowchart.

A total of 14 participants were included in this study. Figure 1 displays the flowchart of the current study.

Participants’ characteristics, including age, height, body mass, body mass index (BMI), sex distribution, educational level, marital status distribution, monthly income distribution based on minimal wage salary, and the number of falls in the last year, are presented in Table 1.

**Table 1.** Participants’ characteristics.

Variables	(*n *= 14)
Age (years)	69.9 ± 7.7
Height (cm)	159.4 ± 10.4
Body mass (kg)	73.5 ± 16.5
BMI (kg/m^2^)	28.9 ± 6.1
Sex distribution	57% (8 females) 43% (6 males)
Educational level (years)	7.6 ± 4.0
Marital status	71.4% (10 married) 28.6% (4 single)
Monthly income (MI)	100% (≤ 3 minimal wage)
Falls in the last year	0%

Values are mean ± SD. *n:* number of participants; BMI: body mass index; MI: monthly income based on minimal wage salary. Participants educational levels ranged from 1 to 16 years of education. No falls were reported in the study.

The repeatability of the self-administered assessments for STS, GST, and TUG was evaluated on two separate occasions, resulting in a total of 28 tests (14 participants × 2 days of testing). All assessments passed the Shapiro-Wilk normality test and the Brown-Forsythe equal variance test (*p *> 0.05). Therefore, a paired T-test was used to determine potential statistical differences between the 2 days of testing. None of the variables showed statistical differences between the 2 days of testing for all assessments (*p *> 0.05). The mean magnitudes of the CV were considered excellent (< 10%) for STS (CV = 4.9%), GST (CV = 2.0%), and TUG (CV = 2.5%). The standard error of the measurements (SEM) was negligible (STS = 0.08; GST = 0.28; TUG = 1.27). The ICC values were excellent (> 0.90) for STS (ICC = 0.97), GST (ICC = 0.98), and TUG (ICC = 0.99) (Table 2). The calculated Cohen's *d* value was −0.14 for STS, 0.10 for GST, and −0.07 for TUG. In the context of test–retest reliability, smaller Cohen's *d* values close to 0 indicate higher stability and reliability.

**Table 2.** Day 1 and day 2 95% confidence interval of the mean difference, coefficient of variation, standard error of measurement, and intraclass correlation coefficients (95% ICC).

Assessments	Day 1	Day 2	95% CI means difference	CV (%)	SEM	ICC (95% ICC)
STS (reps)	9.5 ± 2.9	9.9 ± 2.9	−0.92–0.06	4.9 ± 5.2	0.08	0.97 (0.91–0.99)
GST (ms^−1^)	0.42 ± 0.1	0.41 ± 0.1	−0.01–0.02	2.0 ± 1.9	0.28	0.98 (0.96–0.99)
TUG (s)	12.0 ± 5.9	12.2 ± 6.5	−0.96–0.08	2.5 ± 3.7	1.27	0.99 (0.99–1.00)

Values are mean ± SD. STS: 30-s sit-to-stand test; reps: repetitions; GST: gait speed test; ms^−1:^ meters.second^−1^; TUG: timed up and go test; s: seconds; CI: confidence interval; CV: coefficient of variation (CV = (SD/mean)*100)); SEM: standard error of measurement (SEM = SD×√1−ICC); and ICC: intraclass correlation coefficient.

**Figure 2.**
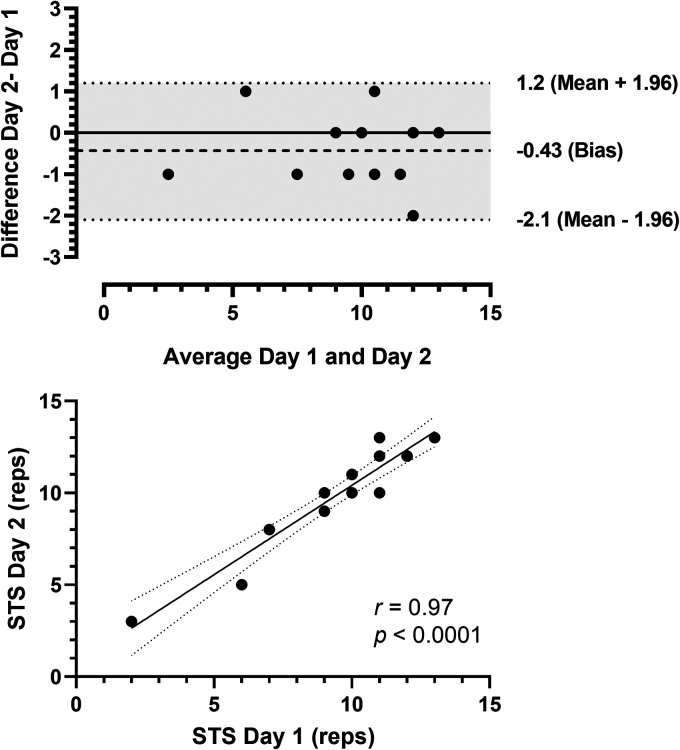
Bland-Altman plots of the 30-second sit-to-stand test (panel A) comparing 2 days of testing (difference assessment = difference day 2 – day 1, average = (day 1 – day 2)/2). Solid horizontal lines show the mean values, and dashed horizontal lines represent the 95% confidence limits. Correlation between the first and second days of the 30-second sit-to-stand test (panel B). STS: 30-second sit-to-stand test; reps: repetitions.

Figure 2 displays the results of the Bland-Altman and Pearson product–moment correlation analyses for STS. The Bland-Altman analysis indicated that the 95% limits of agreement between the 2 days of testing ranged from −2.1 to 1.2, with a bias of −0.4. The data also showed no significant deviation of the mean value from 0 and relatively small variability about the mean (Figure 2A). Furthermore, the correlation analysis revealed a statistically significant, very strong positive correlation (*r *≥ 0.90) between days of testing (*r *= 0.97, *p *< 0.0001, Figure 2B).

**Figure 3.**
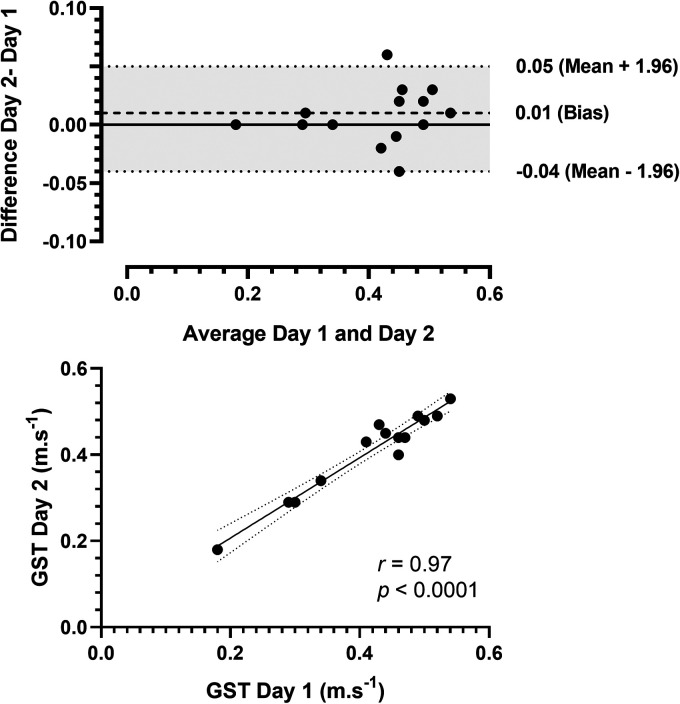
Bland-Altman plots of gait speed test (panel A) comparing 2 days of testing (difference assessment = difference day 2 – day 1, average = (day 1 – day 2)/2). Solid horizontal lines show the mean values, and dashed horizontal lines represent the 95% confidence limits. Correlation between the first and second days of the gait speed tests (panel B). GST: gait speed test.

Figure 3 displays the Bland-Altman and correlation analysis results for GST. The Bland-Altman analysis indicated that the 95% limits of agreement between the two days of testing ranged from −0.04 to 0.05, with a bias of 0.01. The data also demonstrated no deviation of the mean value from 0 and relatively small variability about the mean (Figure 3A). Additionally, the correlation analysis showed a statistically significant, very strong positive correlation (*r *≥ 0.90) between days of testing (*r *= 0.97, *p *< 0.0001, Figure 3B).

**Figure 4.**
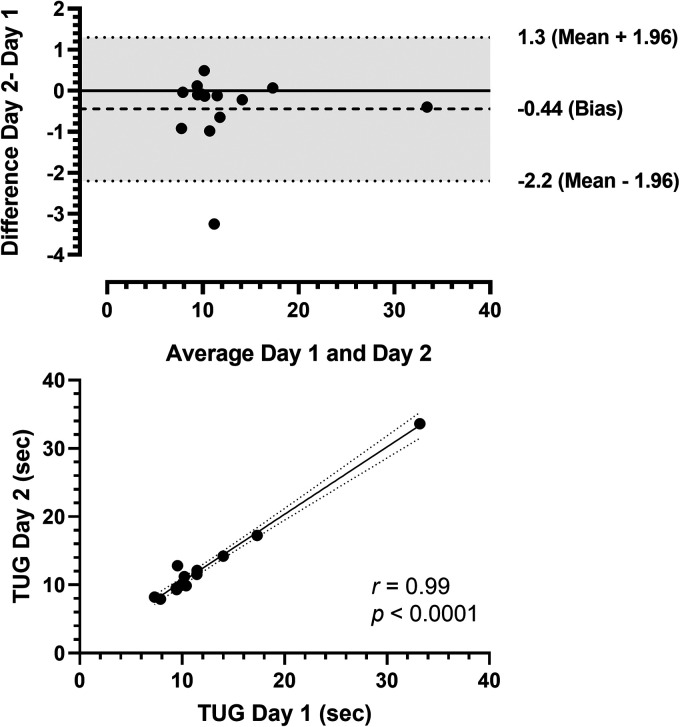
Bland-Altman plots of timed-up and go test (panel A) comparing 2 days of testing (difference assessment = difference day 2 – day 1, average = (day 1 – day 2)/2). Solid horizontal lines show the mean values, and dashed horizontal lines represent the 95% confidence limits. Correlation between the first and second days of the timed-up and go tests (panel B). TUG: timed-up and go.

Figure 4 displays the results of the Bland-Altman and correlation analyses for TUG. The Bland-Altman analysis demonstrated that the 95% limits of agreement between the two days of testing ranged from −2.2 to 1.3, with a bias of −0.4. The data also showed no deviation of the mean value from 0 and relatively small variability about the mean (Figure 4A). Moreover, the correlation analysis indicated a statistically significant, very strong positive correlation (*r *≥ 0.90) between days of testing (*r *= 0.99, *p *< 0.0001, Figure 4B).

## Discussion

This study aimed to determine the test-retest reliability and agreement of home-based self-administered functional capacity assessments in socially isolated older adults. The main findings of the current study provide evidence that the self-administered assessment for STS, GST, and TUG are reliable and in good agreement. Thus, they are suitable for assessing the functional capacity of older adults in home settings, as anticipated in our hypotheses. We showed that these specific, simple, short-duration, low-cost assessments, requiring minimal specialized equipment, can be self-administered remotely and synchronously at home by older adults without in-person supervision, which aligns well with the Peyrusqué et al. study.^13^

### Self-administered assessments

The self-administered assessments for STS, GST, and TUG performed at home showed reliability and good agreement between self-testing days. There were no statistically significant differences between days of testing (*p > *0.05). The mean CV was considered very good (∼ 5%), range: 2.0%–4.9%), with a small SEM, excellent ICC, and strong correlations for all tests (Table 2). The Bland-Altman plots (Figure 2A to C) indicated good agreement. In the present study, the home-based self-administered assessments proved feasible and safe, as all participants could understand and perform the tests without reporting any adverse events to the researchers. Previous studies have shown that supervised functional capacity assessments are reliable in clinical and home settings. Recently, remote assessment applications have been demonstrated in various populations, including people living with cancer,^17^ those with mobility disorders,^14^ individuals with mental health conditions,^15^ people living with type 2 diabetes,^16^ and community-dwelling older adults^11–13^.

Although there is limited scientific literature on this topic, the results of the present study are supported by recent research in community-dwelling older adults.^13,18,20^ Peyrusqué et al.^13^ and Buckinx et al.^20^ reported that web-based video conferencing for remote assessments proved feasible, reliable, and valid compared to in-person physical performance assessments in community-dwelling older adults. The sample size in Peyrusqué et al.^13^ (*n *= 15) was very close to our participants’ sample (*n *= 14), and our main findings are consistent.

In the Peyrusqué et al.^13^ study, ICC values ranged from 0.62 to 0.99, SEM ranged from 0.11 to 7.80, and MDC ranged from 0.30 to 21.6. The ICC value for STS was similar between studies (0.97), while the ICC for TUG and GST were higher in our study (TUG = 0.99; GST = 0.98) compared to Peyrusqué et al.^13^ (TUG = 0.83; GST 0.77). The SEM values reported for STS (0.08), GST (0.28), and TUG (1.27) in the present study are in a similar range to the values described by Peyrusqué et al.^13^ (STS = 1.76; GST = 0.11, and TUG = 0.56). In the Buckinx et al.^20^ study, intra-rater ICC values for the STS ranged from 0.87 to 0.96, SEM of 1.0 (8.0%), and MDC of 0.43 (3.3%). For GST, ICC values ranged from 0.93 to 0.98, SEM of 0.75 (12.3%), and MDC of 0.31 (5%). For TUG, ICC values ranged from 0.91 to 0.97, SEM of 0.85 (7.7%), and MDC of 0.35 (3.2%). These values are close to the ranges reported in the current study. Web-based technologies, including gerontechnology, used at home, whether asynchronous or synchronous, have proven feasible and acceptable alternatives for community-dwelling older adults.^16,18,20^

### Limitations

However, the limitations of the current study need to be addressed. The sample size is small, which may limit the generalizability of the results. Despite demonstrating reliability, some variability still exists in the self-administered assessments. Various factors could contribute to data variability. Despite sending video instructions and confirming distances with participants during test setup, slight discrepancies in measured distances cannot be ruled out. Additionally, potential delays caused by unstable internet connections may affect the precision of remote synchronous assessment, although not noticeable by the researcher during data collection. Despite instructing participants to position their cameras at angles that allowed clear visualization of the test start and end points, measurement errors resulting from camera positioning cannot be entirely excluded.

To mitigate variability, participants received simple, specific, concise, and objective instructions via videoconferencing and instructional videos in advance, explaining how to set up and perform the tests. Participants were also encouraged to contact the researcher before testing if they had any questions or concerns or needed further clarification. Furthermore, the tests chosen for this study were selected with the specific needs of the target population (older adults) in mind. We selected tests that were relatively easy to understand and execute, had short durations, and required minimal. Participants were familiarized with the equipment used for testing (e.g. measurement tape and tape) to minimize variations.

It is important to acknowledge the potential effects of ceiling and learning when assessing the test–retest reliability of the STS, GTS, and TUG. To mitigate ceiling and learning effects, the gerontologist researcher was trained to perform the tests consistently, participants received standardized instructions and instructional videos, were provided with familiarization before the tests, and the order of tests was randomized and counterbalanced across participants. Additionally, sufficient recovery was provided between test–retest sessions (4 weeks apart) to avoid potential fatigue effects. Since no statistically significant differences were found between the days of testing, these procedures were deemed successful, indicating that learning effects are unlikely. Although we took several precautions to mitigate ceiling effects, their presence cannot be completely ruled out.

### Practical Applications

Amid the COVID-19 pandemic, with its associated restrictions and social isolation measures, there was a pressing need for simple and reliable self-administered functional capacity assessments, particularly for older adults. Such assessments are critical for people living in long-term care facilities, at home alone, and in hospitals. With minimal equipment requirements and easy-to-follow instructions, these assessments offer a cost-effective and accessible way of monitoring older adults’ health and functional capacity, particularly those with limited musculoskeletal strength, endurance, and mobility.

Furthermore, reliable self-administered functional capacity assessments conducted remotely and synchronously at home serve as clinically significant diagnostic tools to be used in clinical practice.^16,20^ They help determine the impact of aging on functional capacity, muscular function, and overall health. Additionally, they enable healthcare professionals to establish baseline information and conduct follow-up assessments without needing in-person visits. This eliminates barriers such as physical limitations, transportation issues, lack of caregivers, waiting periods, fatigue, stress, weather-related challenges, and health conditions.^13^ Remote assessments can also be used to support healthcare professionals in monitoring the effectiveness of preventive and rehabilitation interventions as well as individualized exercise programs, optimizing treatment outcomes.^46^

Recent studies suggest that web-based video conferences are valid for assessing functional capacity^13,16,18^ and assessing the efficacy of supervised home-based, real-time videoconferencing in patients with type 2 diabetes.^47^ These results support the implementation of telehealth as an alternative for rehabilitation practices.^47^ In a scoping review, Sanchez-Ramirez et al.^48^ reported that patients who received telemonitoring and/or telerehabilitation experienced improvements in exercise capacity, enhanced health-related quality of life, and reduced healthcare services utilization, which aligns well with the findings of Karanasiou et al.^47^

As telemonitoring and telerehabilitation have demonstrated positive impacts and appear as effective as standard care^20,47,48^ incorporating self-administered assessments into health monitoring is prudent and necessary. Older adults, particularly those facing social isolation, and healthcare professionals should take advantage of simple remote self-assessments to determine their functional capacity and health status. Nevertheless, home setting limitations need to be considered, including the available space for testing, safety concerns, participants’ access to and proficiency in using the internet and web-based conference software, and the precise replication of the test setups.^13^

The COVID-19 pandemic highlights the need for further research on remote testing, especially in socially isolated older adults and other populations. Rigorously conducted reliability and agreement studies are essential in this regard.^22^ The association of functional capacity tests with the ambulatory assessment of physical activity or exercise intervention program effectiveness, alongside the development of new assessment strategies, should push the boundaries of how people will be assessed and treated in the future. The continuous advancement of technology and healthcare provides opportunities for integrating remote assessments as a promising tool for improving patient care and expanding access to healthcare services.^20^

Since aging is associated with a greater risk of falls, falls-induced injuries, chronic diseases, musculoskeletal disorders, hospitalizations, morbidity, and mortality,^49–51^ physical activity becomes critical for successful healthy aging. Studies have consistently shown that physical activity plays a significant role in improving physical fitness, functional capacity, functional mobility and balance, and musculoskeletal function, decreasing the risk of falls, chronic diseases, and pain in older adults.^46,52–54^ Such evidence emphasizes the importance of functional and physical fitness assessments to capture the benefits of physical activity for older adults. These assessments are fundamental in quantifying improvements, identifying weaknesses, and tailoring exercise programs to meet persons’ needs.

Future studies should target expanding participant sample size to enhance the generalizability of these findings. New investigations should also confirm the reliability of self-administered measurements performed by the participants compared to a trained researcher rater using automated video processing tools. Studies comparing remote and traditional in-person assessments and investigations into their applicability in diverse conditions, such as frailty, chronic diseases, and metabolic syndrome, should be further explored. Sensor-based assessments of community-dwelling older adults should be further explored in the context of remote assessments.^55^ These studies will shed more light on the potential of self-administered assessments at home to improve older adults’ healthcare and well-being, health, and quality of life.

## Conclusions

In conclusion, the self-administered assessments for the STS test, GST, and TUG test, conducted remotely and synchronously by community-dwelling older adults without in-person professional supervision, consistently demonstrated high reliability. These assessments exhibited low coefficients of variation, averaging ∼ 5%, and excellent intraclass correlations ranging from 0.97 to 0.99, accompanied by strong positive correlations, and minimal standard measurement error values. These results strongly support the use of self-administered STS, GST, and TUG assessments at home without needing in-person professional supervision among community-dwelling older adults.
